# On the Therapeutic Value of Alkaline and Earthy Sulphites in the Treatment of Catalytic Diseases

**Published:** 1865-01

**Authors:** H. R. De Ricci


### Selections.

### ON THE THERAPEUTIC VALUE OF ALKALINE AND EARTHY SULPHITES IN THE TREATMENT OF CATALYTIC DISEASES.

By Dr. H. R. De Ricci.

[The discovery, if indeed it may be so called, of the action of sulphites in certain forms of catalytic (zymotic) diseases, was made by Dr. Polli of Milan. Since the publication of Dr. Polli's papers on the subject, Dr. De Ricci has devoted much time and attention to its investigation.]

For the benefit of those who may not have read my former publications on the action of the alkaline and earthy sulphites, nor the original writings of Professor Polli, I shall now, as briefly as possible, recount the origin and result of his discoveries.

For several years past, a theory has been almost universally gaining ground that a great number of diseases depend essentially on the presence of an organic poison circulating in the system, where, acting as a ferment in the blood, it multiplies itself, vitiating the animal fluids and giving rise to divers diseases, according to the special poison in circulation; thus in one case producing small-pox, in another scarlatina, in another puerperal fever, &c. These diseases have been termed *zymotic*, on the supposition that the circulating poison acted as a *ferment* in the blood; but, as the precise mode of action of these animal poisons is not as yet well understood (though it is no doubt a chemicovital action), I shall throughout this paper employ the term *catalytic* instead of *zymotic*, as perhaps better calculated to convey the idea of the function of these poisons, without absolutely involving a principal, which the term zymotic might do.

Several years ago, Professor Polli, being fully convinced of the truth of the catalytic theory of many diseases, devoted his talents to search out some means by which these poisons, even after they were absorbed and developed in the animal economy, might be neutralized, and eventually eliminated, without, at the same time, imperilling the integrity of the blood. The task was an arduous one, for the great physiologist, C. Bernard, had dogmatically asserted, that any substance capable of destroying a catalytic poison in the blood would, at the same time, so alter that fluid itself that it would be rendered incapable of performing its vital functions. Professor Polli, however, had long observed the action of sulphurous acid in arresting the process of fermentation. He was convinced in his own mind that in it would be found the anti-catalytic substance he was seeking for. He persisted in his researches, and eventually succeeded in establishing that not only sulphurous acid possesses the property of arresting fermentation and neutralizing catalytic action, but that also its alkaline and earthy compounds have the same power, with this difference, that while sulphurous acid cannot in any way be introduced into the circulation with safety, the alkaline and earthy sulphites, hyposulthites and bisulphites can be administered with the greatest impunity, even in large doses; and their presence can always be detected when administered, not only in the blood but in the tissues and evacuations. Having satisfactorily established this important fact, he began a series of experiments on animals, which proved, most successfully, the correctness of his theory and the accuracy of his deductions. Nothing could give me greater pleasure than to recount the several interesting experiments which he performed, but it would lengthen this paper too much, and otherwise occupy too much of the space of the Journal; and, for those who are interested in this matter, I beg to refer them to a communication read by me, about a year ago, at the Obstetrical Society in this city, in which will be found a short epitome of Professor Polli's experiments.

From the moment I first read the account of Professor Polli's researches I came to the conclusion that his discovery was either valueless or of immense value. In such a case there could be no medium; it was either utterly worthless, or it was a discovery second to none—a discovery even greater than that of vaccination, and one which would constitute one of the greatest epochs in the annals of medicine; for vaccine could, at most, only ensure the vaccinated against one single disease, while the sulphites—if they truly could neutralize the action of catalytic poisons—of such poisons as *glanders* (see Polli's experiments)—would be able to arrest the course of small-pox, hydrophobia, syphilis, infection from dissecting wounds, erysipelas, puerperal fever, measles, scarlatina, whooping-cough, dysentry, diarrhœa, cholera, influenza, typhus, dothinenteria, plague, diphtheria, &c., &c., all of which are classed by Dr. Farre as zymotic diseases. The question seemed to me of such vast magnitude and importance that I solicited in every direction for co-operation in testing the real value of these sulphites. I first tried them on myself, and finding them decidedly harmless, even in doses of one scruple, five or six times a day, I began to administer them in every case where I thought that a catalytic poison might be the cause of disease. In no case did I meet with any mishap, and in most cases I think I can fairly say I was successful. In no case did the exhibition of the sulphites produce sickness of stomach, diarrhœa, or any other inconvenience; and in every case their administration seemed to be of decided advantage.

The first case I shall describe is one of great interest and value in a clinical point of view; it was undoubtedly a case of infection from an animal poison. The patient was almost given over as lost, so severe were the symptoms of the disorder; yet the patient recovered, and the treatment consisted solely in the administration of the bisulphite of soda, in full, repeated, and continued doses.

A lady of about forty-five years of age, of sound constitution, and in the enjoyment of excellent health, was suddenly called, about a year ago, to the death-bed of one who was very dear to her. That death-bed was fearfully sudden and unexpected, and that poor lady could not be persuaded, long after death had indubitably taken place, that the spirit of the beloved one had really fled. She would not leave the corpse; she threw herself on it, and kissed it over and over again, and could not be induced to leave it, even when the discoloration of the skin and the offensive smell of rapidly-advancing decomposition gave ample testimony of the reality of death. The burial was performed two days after death, owing to the rapid decomposition of the body; and, soon after the funeral, I was hastily summoned to the bedside of this lady, whom I found in the following condition. It was about five in the morning when I entered the bedroom. She had gone to bed the night before quite calm and resigned, and on the previous day she had partaken fairly of food, but had not eaten any thing which could in any way account for the state in which I found her, of which the following is a fair sketch:—The windows of the bedroom being open, the morning light streamed freely into the room, and as I approached the foot of the bed I had a full view of the patient's face. To those of my readers who, like myself, have been familiar with the victims of cholera, I shall simply say that the patient looked like one in cholera, in the stage of collapse. To those who have not yet had the melancholy opportunity of witnessing a case of that terrible disease, I shall say that I hardly recognized my friend, so altered, and pinched, and ghastly were her features. Her eyes were sunk, and surrounded by a lead-colored zone; her cheeks, which, but a day before, were plump and ruddy, were now hollow and sunk; her eyes were glassy; her pulse scarcely to be felt; the surface of the body cold; her breath cold; her tongue cold; her voice low and husky; her faculties perfectly clear. She now lay quite prostrate on her back; but for some hours previously had suffered greatly from vomiting, cramps, and diarrhœa, which was of the characteristic rice-water appearance always observed in cholera. I at once administered a full supply of hot brandy and water, ordered turpentine stupes to the abdomen and limbs, treating the case exactly as one of Asiatic cholera; and, being fully alive to the grave nature of the attack, requested for further advice, and at once sent off for my valued friend, the late Dr. Mayne. At first I felt singularly at a loss how to account for so sudden and severe an attack of what seemed to be a genuine case of Asiatic cholera. So true was it to symptoms that, when Dr. Mayne looked at the patient, he whispered to me: "If cholera were in the country one would not hesitate to give this case a name." The clue to the disease was, however, given to me, in a few words, by the patient herself. As soon as she had taken the brandy she said to me:—"The smell of the body was dreadful; I cannot get rid of it in any way;" and immediately she began to retch. She had given me the key, and the mystery of her case was solved. It was no doubt a case of putrid infection—of septicemia; and if there was any truth in the "sulphite" theory it should prove of value in such a case as this. I at once ordered a strong solution of bisulphite of soda in infusion of quassia, with tincture of bitter orange peel and Battley's sedative—two drachms of the bisulphite to the ounce—and give it in large teaspoonful doses, every half hour at first, and then every hour, each dose containing nearly twenty grains of the bisulphite. I watched this case incessantly myself, day and night, and the result was most satisfactory. All the symptoms by degrees abated, and in a very few days the patient was fully convalescent. Dr. Mayne, who had anxiously watched this case with myself, was so impressed with the results obtained that he told me he would give the remedy a full trial in every case of scarlatina that should come under his care; and, in addition, promised (at my suggestion) that he would prescribe a dose of the sulphites daily to every healthy member of the family in which there was a case of scarlatina, to test its value as a prophylactic; for if a sulphite could destroy a catalytic principle, even when developed in the system, it should also have the power of preventing the development of that principle from the commencement.

As I stated above, the lady recovered, and, apparently, was restored to absolute health; she, however, complained to me occasionally of wandering pain, and general *mal-aise* at times, which she could not account for. About five months after her recovery she hurt her leg; it was a very trifling abrasion, but it assumed an angry look, and seemed determined not to heal, when at the end of two months she broke out all over with an extraordinary eruption, more like erythema-nodosum than anything else, when I at once placed her again on bisulphate of soda, in the supposition that some of the poison was still lurking in her blood, when the sore in her leg rapidly healed and the eruption disappeared, leaving the patient perfectly well.

The next two cases in my list were well-marked types of measles. I shall not take up the time of my readers by giving the details of them. I shall merely state that they were severe cases; that they were treated solely with bisulphite of soda, in scruple doses, every second or third hour, and that both cases grew rapidly well.

The fourth case is one of poisoned wound. A. B., aged about thirty-five, a gardener, was grafting a cactus, and in carelessly handling such a thorny plant, got the back of his left hand severely stung in different places. He plucked out as many of the spines as he could discover, and thought no more about the matter; but in the course of twelve hours or so the hand began to swell and be painful. He at once wrapped it up in a poultice; but the pain and swelling not abating, his employer sent him to me three days after he had been stung. I found his hand enormously swelled, of a dusky purple color, with large bullæ over the dorsum; the forearm also swelled, though not discolored, but presenting several longitudinal red lines running up to the elbow. The man complained of great pain locally, of intense thirst, headache, loss of appetite, shivering, and general feeling of sickness. His tongue was furred and brown. He had, of his own accord, taken a dose of senna and salts that morning. I at once made an incision in the dorsum of the hand, and some pus came out. It was not, however, like cutting into an abscess—no gushing of matter took place, only an oozing, like as if I had cut into a sponge saturated with pus. I therefore made a second incision, parallel to the first, from which some more pus came out; ordered him to wrap up the hand in a large linseed-meal poultice, and prescribed the bisulphite of soda, in scruple doses, every second hour. I desired the man to keep quiet at home—without, however, ordering him to keep his bed; directed him to take light nutritious food, and to drink two pints of XX porter in the day. The following day the hand looked better; it was less swollen and purple, and the pus was in larger quantity and better looking. The man felt also better; he had less thirst, and had only had two slight shiverings. The bowels not having been freed, I ordered him some sulphur, with magnesia and scammony, as an aperient, and to persist with the bisulphite, taking it now every third hour. The following day the man was so much improved that he called on me. The hand looked still better; the incisions which I had made had ulcerated somewhat round their edges, but the suppuration was free, and the pus seemed quite healthy; there was no pain in the arm, and the red lines were very much paler. Matters looking so very much better, I desired the man to take the sulphite only three times in the twenty-four hours; to take a good allowance of food; to stay much in the open air; but to still keep the arm in a sling, and the hand wrapped in a poultice. Two days later he again came; when, finding that all swelling had subsided, all pain gone, all red lines disappeared, the sore inclined to granulate, I stopped the poultice, and desired him to dress the hand with some warm dressing, containing a little balsam of Peru, and to take one scruple of the bisulphite twice a day. Two days after the man returned to me not looking as well as at the previous visit. He had a yellowish tinge in his skin; his tongue was foul; he complained of chilliness, almost amounting to shivering, and the sore at the back of the hand looked unhealthy and angry. On stripping the arm, the red lines were again visible, but of a very pale red; while above the elbow, from its bend to the axilla, a hard, knotty, and extremely painful cord could be felt and seen running up parallel to the brachial artery. I at once returned to the primary doses of the bisulphite, wrapped up the hand in a poultice, and watched to see what the result would be. The man immediately began to mend, and in four days not a trace of hardness was to be felt. Fearing, however, a relapse, I continued administering four scruples of bisulphite daily for ten days longer, by which time the hand was perfectly healed, and the man returned to his work.

Two better cases for testing the effects of sulphites could not possibly have been selected; in both cases the disease clearly resulted from the working of a poisonous element in the blood, evidently introduced from without in the first case, whilst it may in the second case have originated within the system subsequent to the stinging with the thorns, if one does not feel justified in considering cactus thorns as poisonous of themselves; be this, however, as it may, the second case was as clearly one of purulent absorption as the first was of putrid infection—the red lines up the arm marking the course of the inflamed lymphatics as the hard knotty condition of the veins denoting the phlebitic inflammation. Both were treated solely with sulphites, and both completely recovered. It would be great presumption, no doubt, to say that in both cases the patients would have lost their lives except for the saving properties of the bisulphite of soda administered; but I still cannot avoid believing that the sulphurous acid did prevent the spreading of the catalytic principle by rendering it incapable of reproducing itself; and, while keeping it in abeyance, allowed time for its elimination by the ordinary powers of nature; and I think we have a confirmation of this view in the occurrence of a relapse in both cases, where it would appear as if, when the bisulphite was stopped, all the poison had not yet been eliminated, the blood disease breaking out again the moment that the poison was freed of its antagonist; and a cure being effected by administering more of the anti-catalytic remedy until every trace of the animal poison was eliminated.—*Dublin Quarterly Journal, August,* 1864, *p.* 28.

